# Supplementary material for: Clinical value of serum DJ-1 in lung adenocarcinoma
Source: PeerJ. 2024 Jan 29;12:e16845. doi: 10.7717/peerj.16845 (PMC10832618; doi:10.7717/peerj.16845)
Supplement: Supplemental Information 2 [file peerj-12-16845-s002.docx]

| **Gene** | **Primer** |
| --- | --- |
| **β-actin** | **(Sense primer)** 5' AGAAAATCTGGCACCACACCT 3' **(Anti-sense primer)** 5' GATAGCACAGCCTGGATAGCA 3' |
|  |  |
| **DJ-1** | **(Sense primer)** 5' AACGCTGAAGCGTCCAGAAT 3' **(Anti-sense primer)** 5' TGACCACATCACGGCTACAC 3' |
|  |  |
